# Spatial expression of *CLAVATA3* in the shoot apical meristem suggests it is not a stem cell marker in soybean

**DOI:** 10.1093/jxb/ert341

**Published:** 2013-10-31

**Authors:** Chui E. Wong, Mohan B. Singh, Prem L. Bhalla

**Affiliations:** Plant Molecular Biology and Biotechnology Laboratory, ARC Centre of Excellence for Integrative Legume Research, Melbourne School of Land and Environment, The University of Melbourne, Parkville, Victoria 3010, Australia

**Keywords:** CLAVATA3, cytokinin, shoot apical meristem, soybean, stem cell.

## Abstract

*CLAVATA3* (*CLV3*), a stem cell marker in *Arabidopsis thaliana*, encodes a secreted peptide that maintains the stem cell population within the shoot apical meristem. This work investigated the *CLV3* orthologue in a major legume crop, soybean (*GmCLV3*). Instead of being expressed in the three outermost layers of the meristem as in *Arabidopsis*, *GmCLV3* was expressed deeper in the central zone beneath the fourth layer (L4) of the meristem, overlapping with the expression of soybean *WUSCHEL*. Subsequent investigation using an alternative stem cell marker (GmLOG1) revealed its expression within layers L2–L4, indicating that GmCLV3 is not a stem cell marker. Overexpression studies of *GmCLV3* in *Arabidopsis* and complementation of *clv3-2* mutant suggest similar functional capacity to that of *Arabidopsis CLV3*. The expression of soybean *CLV1*, which encodes a receptor for CLV3 in *Arabidopsis*, was not detectable in the central zone of the meristem via reverse-transcription PCR analysis of amplified RNA from laser-microdissected samples or *in situ*, implicating a diverged pathway in soybean. This study also reports the novel expression of *GmLOG1* in initials of axillary meristem in the boundary region between the SAM and developing leaf primordia, before the expression of *GmWUS* or *GmCLV3*, indicating cytokinin as one of the earliest signals in initiating and specifying the stem cell population.

## Introduction

Plant peptide ligands are signalling molecules that play vital roles in various aspects of plant growth and development. Among them is the CLAVATA3/embryo-surrounding region (CLE) family of peptide, which is characterized by an N-terminal signal peptide and a conserved 14 residues domain (CLE motif) at the C-terminus (Cock and McCormick, 2001). CLAVATA3 (CLV3) from *Arabidopsis thaliana* represents the best-understood member of the CLE family, with the CLV3 ligand known to function in maintaining the stem cell population within the shoot apical meristem (SAM).

Stem cells residing in the SAM are the source of new cells for the development of all aboveground organs. They can self-maintain as well as give rise to daughter cells that leave the stem cell niche to develop into specialized cell type. The SAM can be divided into two distinct regions: the surface tunica layer where cells divide anticlinally resulting in an expansion of the surface area and the underlying corpus consisting of cells that divide in all planes increasing the volume of the apex (reviewed by Steeves, 2006). The tunica comprises of one to five clonal layers (L1–L5) with one layer found in monocot. In addition to this layered organization, the SAM can also be divided into three zones of distinct functions: (i) the central zone that contains stem cells; (ii) the surrounding peripheral zone; and (iii) the underlying rib zone where the initiation of lateral organs and the central stem tissue take place. The rate of cell division in the central zone is much lower than that in the peripheral zone, and stem cells can be distinguished morphologically by a large nucleus with dense cytoplasm and the lack of a large central vacuole.

In *Arabidopsis*, *CLV3* is known to be expressed in the three outermost layers of the central zone (L1–L3) acting in a feedback loop involving CLV1 and a homeobox transcription factor, WUSCHEL (WUS), to regulate the dynamic balance between the two activities of stem cells, proliferation and differentiation. The *CLV3* gene acts as a negative regulator for stem cell proliferation (Clark *et al.*, 1995; Fletcher *et al.*, 1999) while *WUS* is expressed in the underlying region in the organizing centre promotes stem cell activity (Mayer *et al.*, 1998). *CLV3* encodes a small extracellular protein that is processed into a ligand of 13 amino acids (Kondo *et al.*, 2006; Ohyama *et al.*, 2009) and *CLV1* encodes a transmembrane receptor kinase expressed primarily in the L3 layer of the SAM (Clark *et al.*, 1997; Jeong *et al.*, 1999). The CLV3 ligand has been demonstrated to bind CLV1 (Ogawa *et al.*, 2008) leading to the downregulation of *WUS* expression. Mutation in *CLV1* or *CLV3* loci thus result in an overproliferation of stems cells in the central zone. Recent study has highlighted the dynamic of the feedback loop as WUS not only activates *CLV3* expression (Schoof *et al.*, 2000), it can also directly repress the expression of *CLV1* and hence negatively modulates the CLV signalling pathway (Busch *et al.*, 2010).

The phytohormone cytokinin has also been shown to be essential in the regulation of stem cell activities (reviewed by Sablowski, 2011). Mutation in *LONELY GUY* (*LOG*) that encodes an enzyme involved in the conversion of inactive cytokinin to its biologically active form in rice resulted in plants with defective SAM (Kurakawa *et al.*, 2007). There is evidence implicating cytokinin in the positioning of the stem cell niche (Leibfried *et al.*, 2005; Gordon *et al.*, 2009). In fact, cytokinin has been reported to activate *WUS* expression while WUS directly represses the transcription of several two-component type-A *ARABIDOPSIS RESPONSE REGULATOR* (*ARR*) genes, which function as negative regulators for cytokinin signalling (Leibfried *et al.*, 2005; Gordon *et al.*, 2009).

The activity at the SAM largely determines the general architecture of a plant. This has implication on the total amount of sunlight intercepted by a plant, which is critical for biomass accumulation. Later in plant development, the reproductive SAM gives rise to flowers that form seeds following fertilization. Having a better understanding of shoot meristem biology in crops therefore offers potential strategies in improving yield to meet the increasing global food demand. Soybean is the largest legume crop in the world responsible for more than 50% of worldwide oilseed production. While *Arabidopsis* is an excellent model system for studying regulatory network governing SAM function, much remains to be uncovered for that of soybean meristem. Furthermore, the translation of fundamental knowledge obtained using the model plant *Arabidopsis* to corresponding processes in legume crop remains a challenge due to obvious vegetative and floral developmental differences.

This study isolated the soybean orthologue of *Arabidopsis CLV3* and characterized its expression in relation to the spatial expression of *GmWUS* and *GmLOG1*. GmCLV3 functional characteristics were further examined through ectopic expression in *Arabidopsis* and *clv3-2* mutant complementation. This study implies a diverged CLV pathway in soybean and also reveals evidence that supports cytokinin as one of the earliest signals in initiating and specifying the stem cell population.

## Materials and methods

### Plant materials and growth conditions

Soybean plants (*Glycine max* L. Merr. cv. Bragg) were grown from seeds in a greenhouse located at the University of Melbourne, Victoria, Australia while *Arabidopsis thaliana* (Col0) and the *clv3-2* mutant line were obtained from the *Arabidopsis* Biological Resource Centre and maintained under long-day conditions (16/8 light/dark 22 °C) in growth chambers.

### RNA extraction and reverse-transcription PCR analysis

Total RNA from different soybean tissues of 10-day-old plants were isolated using Qiagen RNeasy Mini Kit with on-column DNAse digestion (Qiagen). Subsequent reverse-transcription PCR (RT-PCR) was carried out using a one-step RT-PCR kit (Qiagen) according to manufacturer’s instructions with 20ng total RNA as template. The PCR amplification step was routinely carried out for 30 cycles.

### Plant vectors and transformation

The full-length *GmCLV3*a gene was amplified by PCR using cDNA derived from SAMs and primers (GmCLV3aBamH1F: 5′-CAGGATCCGATCTTCACCACACAACATTAC-3′; GmCLV3aXho1R: 5′-TAGCTCGAGGCCATAAGCTGGTAGAT GTTC-3′). The amplified fragment was subcloned into pENTR1A vector (Invitrogen) at the *Bam*H1 and *Xho*1 site. Following verification of the cloned gene via sequencing, the fragment was transferred into pB7WG2 vector via LR-mediated recombination reaction (Invitrogen). The construct was then transferred into *Arabidopsis* via *Agrobacterium tumefaciens* AGL1-mediated floral-dip transformation method (Clough and Bent, 1998). Primary transformants were screened on soil saturated with 40 µg l^–1^ of the herbicide glufosinate ammonium.

### Laser microdissection of central zone

Dissected soybean shoot apexes were fixed in Farmer’s solution (ethanol/acetic acid 75:25) for 4h on ice. Tissues were then dehydrated through ethanol series starting with 75% ethanol and processed according to Wong *et al.* (2012). Paraffin blocks were sectioned into 10-µm thickness before being stretched on prewarmed diethyl pyrocarbonate-treated H_2_O bath (37 °C) and placed on PALM membrane slides (1mm PEN, P.A.L.M., Bernried, Germany). Slides were then dried for 3h at 37 °C and stored at 4 °C until laser microdissection. Immediately before laser microdissection, slides were deparaffinized in Histochoice (Sigma). The PALM Robot Microbeam laser microdissection system was used for collecting cells from the central zone of meristem. Cells were first selected using the graphics tools of the P.A.L.M. RoboSoftware and then isolated by the laser microbeam. The triangular region presumed to be the central zone was then collected by laser pressure catapulting into the lid of a 0.5-ml microfuge tube placed in a holder closely above the slide. RNA was then extracted and amplified using PicoPure kit and AmbionMessageAmp III RNA amplification kit, respectively, according to manufacturer’s instructions.

### *In situ* hybridization analysis

The soybean shoot apices from 10-day-old plants were dissected and fixed with 4% paraformaldehyde in phosphate-buffered saline overnight at 4 °C following vacuum infiltration. The tissue was then dehydrated through a series of ethanol solution and embedded in paraplast (Sigma, St Louis, MO, USA) following standard methods. The *in situ* hybridization was carried out according to modified protocols from Jackson (1991) as described in Haerizadeh *et al.* (2009). Digoxigenin-labelled antisense RNA probes were transcribed from T7 or SP6 promoter of pGEMT-Easy vector (Promega, Madison, WI, USA) using the DIG RNA Labeling Kit (Roche Diagnostics, Indianapolis, IN, USA) according to manufacturer’s instructions. All hybridization results were observed and photographed using a BX60 microscope and DP70 digital camera (Olympus, Centre Valley, PA, USA).

## Results

### Identification of soybean *CLV3*-like genes

Though soybean genome has recently been sequenced, no *CLV3*-like orthologues could be identified in the predicted Glyma1.0 gene set (Schmutz *et al.*, 2010). When searches were performed using existing *CLV3* sequences against the soybean genome sequence, two matching regions were identified where no gene was annotated. These regions were subjected to further gene prediction analysis at FGENESH (http://www.softberrry.com) and two potential *CLV3*-like transcripts were identified (Gm12:34,928,570..34,929,240 and Gm13:39,688,331..39,688,917). The presence of two *CLV3*-like transcripts in soybean genome is a result of the more recent whole genome duplication event (Schmutz *et al.*, 2010).

**Fig. 1.**
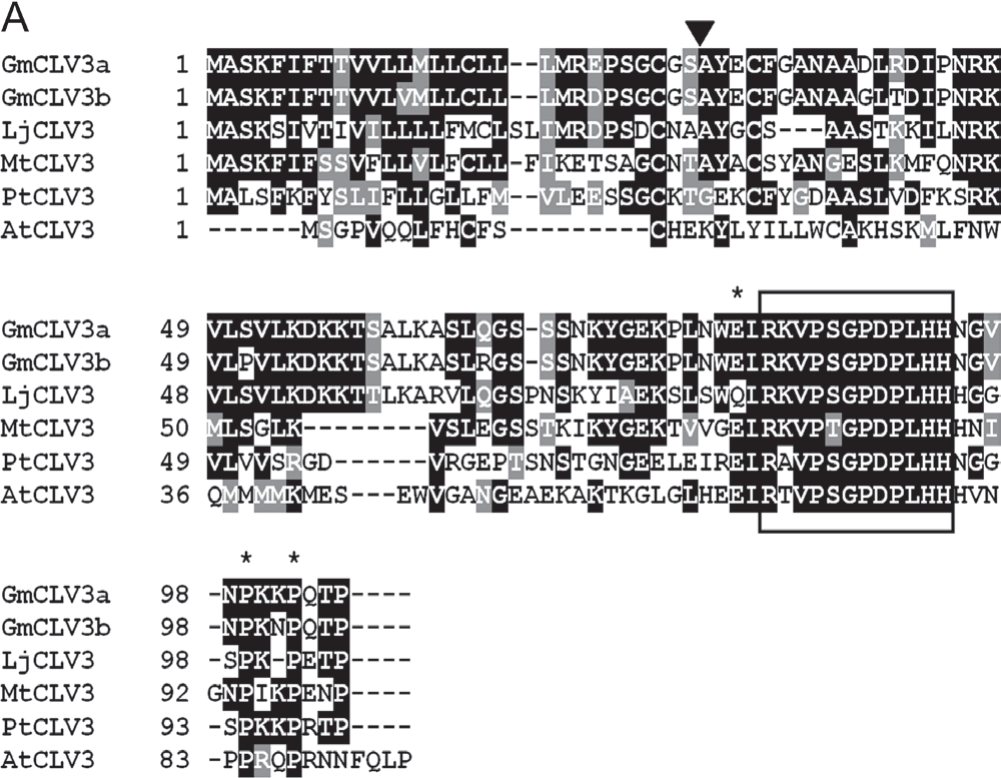

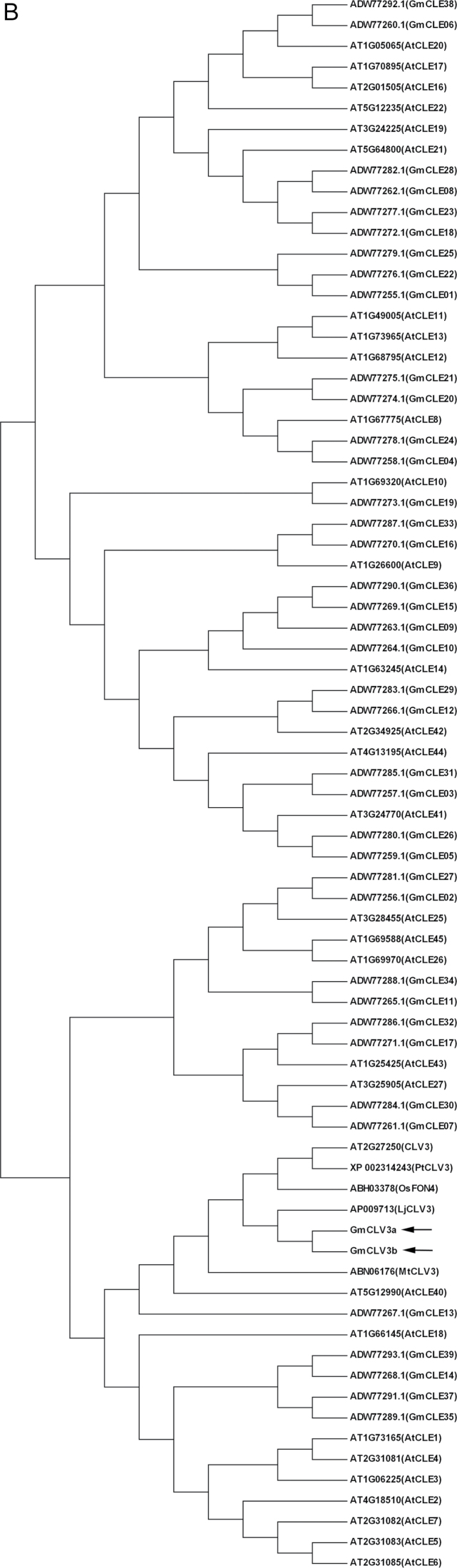
Sequence alignment analysis of GmCLV3. (A) Predicted protein sequences for GmCLV3a and GmCLV3b were aligned with other existing CLV3 orthologues using CLUSTALW (Larkin *et al.*, 2007) and the output was displayed with Box Shade (http://www.ch.embnet.org/software/BOX_form.html). The predicted cleavage site for the signal peptide of GmCLV3s is indicated with a triangle. The 12-amino acid CLE motif is boxed. The three amino acid residues that are part of the secondary conserved motif adjacent to the CLE motif specific to the CLV3 group (Oelkers *et al.*, 2008) are marked with asterisks. AtCLV3, *Arabidopsis thaliana* CLV3 (At2g27250); LiCLV3, *Lotus japonicus* CLV3 (AP009713); MtCLV3, *Medicago truncatula* CLV3 (ABN06176); PtCLV3, *Populus trichocarpa* CLV3 (XP_002314243). (B) Alignment analysis of the CLE domain encoded by all CLE members reported to date for soybean and *Arabidopsis* indicating GmCLV3a and GmCLV3b (marked with arrowheads) are orthologous to *Arabidopsis* CLV3. (Figure is overleaf).

As with *Arabidopsis CLV3*, both *GmCLV3* genes also consist of three exons. They encode small proteins of 105 amino acids in length with a signal peptide predicted at the N-terminal region between amino acids 29 and 30 (Dyrløv Bendtsen *et al.*, 2004). A recent study has divided the CLE family into 13 groups based on sequence similarity at the C-terminal region (12–18 amino acids) encompassing the CLE domain (Oelkers *et al.*, 2008). *Arabidopsis* CLV3 clustered in group 3 and, in addition to the CLE motif, members of this cluster also contains a conserved secondary motif adjacent to the CLE residues (Oelkers *et al.*, 2008). When the virtually translated amino acid sequences of the gene pair were aligned with known full-length dicot CLV3 protein sequences, the highly conserved CLE motif as well as the secondary motif specific to group 3 can be identified (Fig. 1A). In fact, the GmCLV3 homologue only differs at one residue (residue 2) in the CLE domain from that of *Arabidopsis* (Fig. 1A). This study also compared the CLE domains of GmCLV3 with those encoded by all members of CLE family identified in soybean and *Arabidopsis*. As shown in Fig. 1B, GmCLV3a and GmCLV3b form a superclade together with CLV3. These features suggest that this homologous pair is the orthologous sequence of *CLV3* in soybean. Hereafter, these are referred to as GmCLV3a (Gm12:34,928,570..34,929,240) and its homologue GmCLV3b (Gm13:39,688,331..39,688,917).

### Expression of *GmCLV3* and *GmCLV1*

**Fig. 2.**
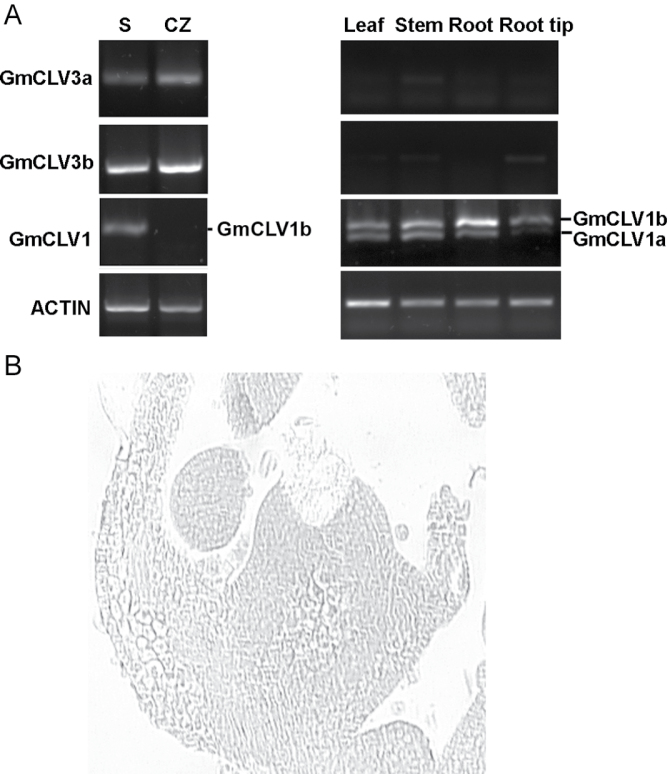
Expression profiles of *GmCLV3* and *GmCLV1* in vegetative soybean tissues. (A) Reverse-transcription PCR was carried out with amplified RNA (left panel) or total RNA (right panel) as templates. S is a sample consisting of amplified RNA derived from one section of a complete shoot apex scratched from the glass slide and CZ is from the central zone. For *GmCLV1* amplification, the lower band (403 bp) corresponds to *GmCLV1a* whereas the upper band (457 bp) represents *GmCLV1b*. *ACTIN* was used as an internal control. (B) One section of the SAM shoot apex after central zone was laser microdissected.

This study investigated the expression pattern of the *GmCLV3* gene pair in different soybean tissues using RT-PCR analysis with primers specific to each of the homologue. To assess their expression in the SAM, laser-microdissection technology was used to obtain amplified RNA from the central zone of the meristem. As expected, both genes were expressed only in the central zone of the SAM but not in other vegetative tissues examined (Fig. 2A).

CLV3 is known to function as a ligand that binds to CLV1 receptor to regulate stem cells’ function (Ogawa 2008). Thus the expression pattern of *GmCLV1* was investigated. The soybean counterparts of CLV1 are annotated in the Glyma1.0 gene set as Glyma11g12190 (GmCLV1a) and Glyma12g04390 (GmCLV1b) and they are the same *GmCLV1* sequences isolated by Yamamoto *et al.* (2000). While *GmCLV1a* function is unknown, *GmCLV1b* corresponds to *GmNARK* that has been reported to play a role in nodulation (Searle *et al.*, 2003) and likely in partnership with members of the CLE family (Reid *et al.*, 2013). The current study proceeded to examine the expression of the *GmCLV1* homologue via RT-PCR analysis (Fig. 2A). *GmCLV1a* transcript was detected throughout vegetative tissues but not in the root tip and central zone of SAM. The expression of *GmCLV1b* is similar to its homologue except its transcripts are also found in the root tip. This expression pattern of *GmCLVb* is largely consistent with that reported by Nontachaiyapoom *et al.* (2007).

### Spatial expression pattern of SAM regulatory genes

**Fig. 3.**
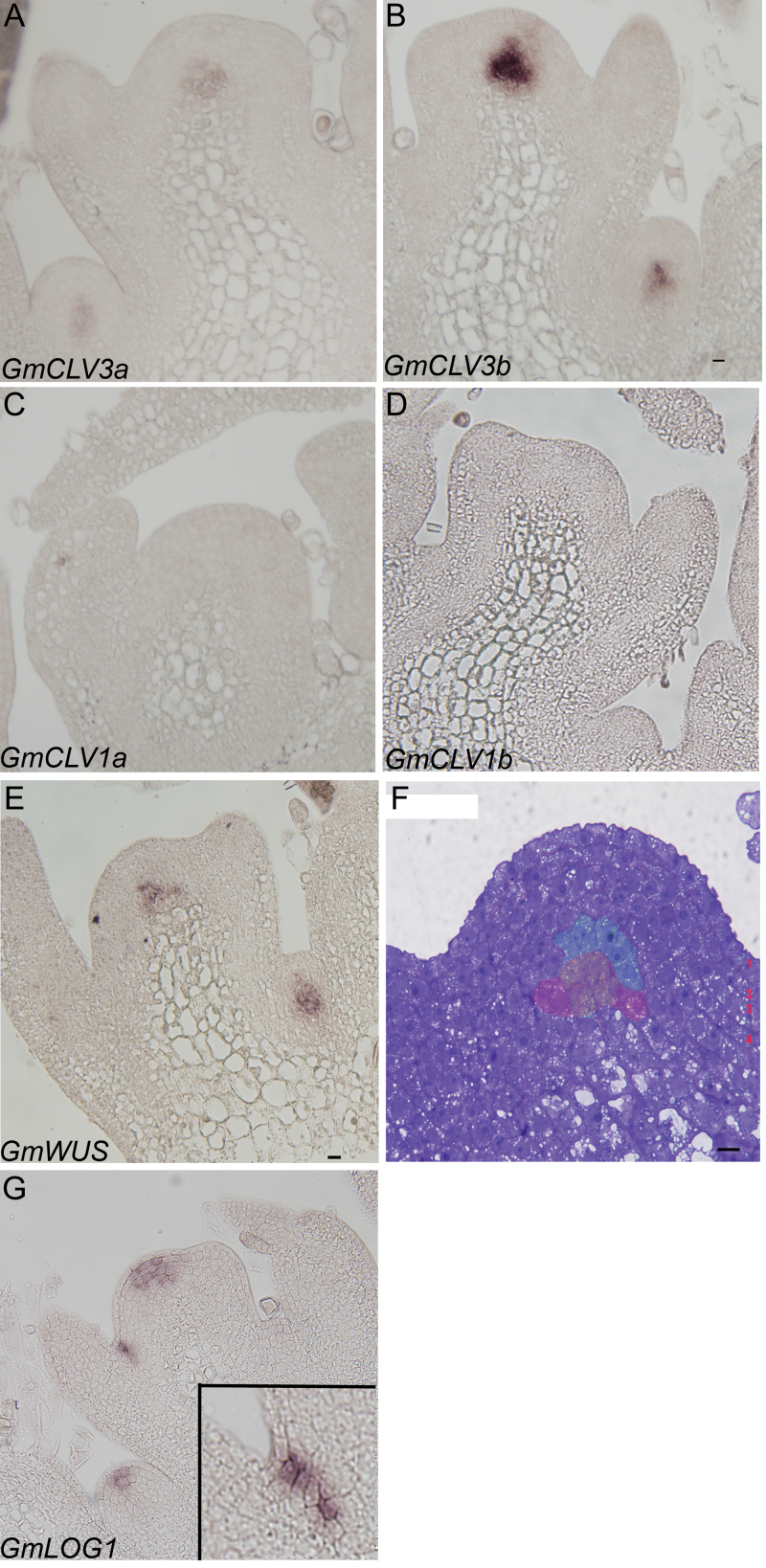
RNA *in situ* hybridization analysis. (A–E and G) Longitudinal sections of soybean shoot apexes hybridized with antisense digoxigenin-labelled RNA as indicated. (A and B) Spatial expression of *GmCLV3a* and *GmCLV3b* are very similar, beneath the fourth layer of the meristem albeit a weaker expression for *GmCLV3a*. (C and D) No signals were detected for either *GmCLV1a* or *GmCLV1b*. (E) *GmWUS* is expressed beneath the fifth outermost layer of the meristem. (F) A longitudinal section of soybean shoot apical meristem stained with toluidine blue with the four distinct zonal layers indicated; the expression domain of *GmCLV3* is false-coloured green (approximately 9 cells in total) while that of *GmWUS* red and the overlapping region yellow. (G) *GmLOG1* transcripts are found with the L2–L4 layer. The inset shows a close up of its expression in the boundary region between the SAM and the developing leaf primordia. Bar, 0 μm (this figure is available in colour at *JXB* online).

When this work examined the spatial localization of *GmCLV3* in the soybean shoot apex, *GmCLV3* was found remarkably to be expressed deeper in the central zone and, more specifically, *GmCLV3* is expressed beneath the fourth layer of the meristem (Fig. 3A and B). This is in stark contrast to *CLV3* expression in *Arabidopsis* whereby *CLV3* is known to be expressed in the first three layers of the meristem, L1, L2, and L3. The expression of *GmCLV3* deeper in the axillary meristem was also observed (Fig. 3A and B). This work also examined the expression of the *GmCLV1* homologous pair in the shoot apex but no signal was observed for either of the gene (Fig. 3C and D) likely due to the absence or low expression level of the gene that is beyond the detection limit of the technique.

*WUS* is known to be expressed in the organizing centre of the SAM. To determine whether the region where the *GmCLV3* was expressed corresponded to the organizing centre and hence the expression of *GmWUS*, the spatial expression pattern for *GmWUS* in the SAM was also examined. Consistent with Wong *et al.* (2011), the expression of *GmWUS* was observed in similar area in the central zone but beneath the fifth layer of the meristem (Fig. 3E). In fact, the *GmWUS* expression domain overlaps with that of *GmCLV3*. When compared to a toluidine-blue-stained section of soybean SAM, *GmCLV3*- or *GmWUS*-expressing cells contain large nuclei with dense cytoplasm and small vacuoles (Fig. 3F). It is also obvious from the section that the tunica consists of four layers indicating soybean SAM has an additional L4 layer, unlike in *Arabidopsis*.

Recently, alternative stem cell markers with *CLV3*-like expression pattern have been reported in *Arabidopsis* and they are *APUM-10* (AT1G35750), *MCT2* (AT5G07930), and *AtLOG1* (AT5G06300) (Aggarwal *et al.*, 2010). To identify location of stem cells in soybean SAM, the spatial expression of corresponding soybean orthologues was interrogated. While this work failed to detect the expression of *GmAPUM-10* (Glyma08g19140) and *GmMCT2* (Glyma18g01600) in the SAM (data not shown), it was successful with *GmLOG1* (Glyma10g09480; Fig. 3G). Based on the expression of *GmLOG1* (Fig. 3G), it is clear that the expression of *GmLOG1* is at least partly confined to the four outermost layer of the meristem except the L1. The absence of *GmLOG1* from the L1 layer can also be seen in the axillary meristem. Intriguingly, *GmLOG1* is also expressed in the boundary region between the SAM and developing leaf primordia (Fig. 3G, inset), likely marking the cells that are competent to initiate axillary meristem in the axils of the primary shoot subsequently. However, no *GmWUS or GmCLV3* transcripts were detected in these initials.

### Constitutive expression of *GmCLV3* promotes consumption of the SAM

This study next examined the functional characteristics of *GmCLV3*. As both GmCLV3 copies are identical in the CLE motif region and since they are expressed only in the SAM during the vegetative development, they likely perform the same functions. Thus, the further analysis focused on the ectopic expression of *GmCLV3a*. The full length *GmCLV3a* cDNA was cloned to be driven by the constitutively active cauliflower mosaic virus 35S promoter and the transgene was introduced into *Arabidopsis* via *Agrobacterium*-mediated floral dip transformation (Clough and Bent, 1998).

**Fig. 4.**
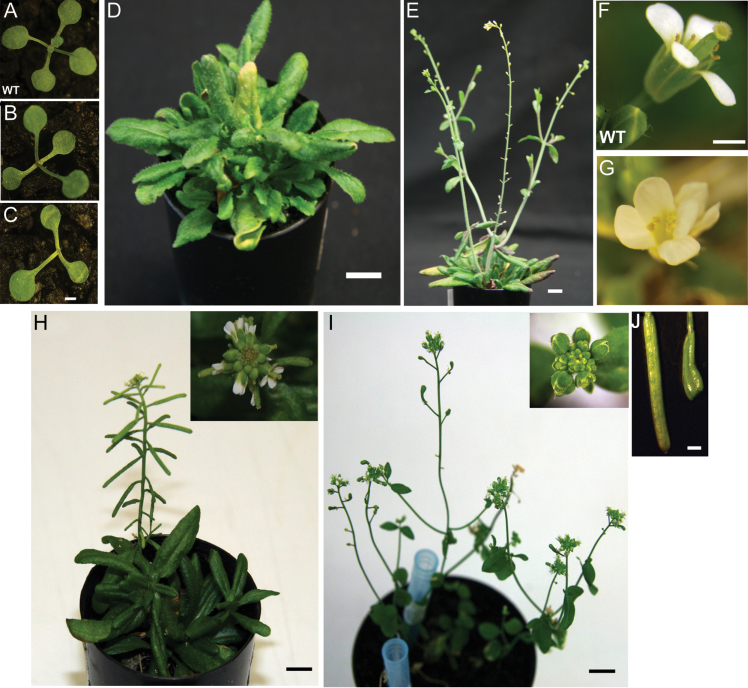
Phenotypes of *35S::GmCLV3 Arabidopsis* plants. (A–C) One-week-old seedlings of wild-type (A) and *35S::GmCLV3* (B and C) displaying a temporary arrest on leaf initiation for plant B while plant C never recovered from the defect. (D) Two-month-old *35S::GmCLV3* plant with leaves formed at irregular phyllotaxis resulting in a ‘bushy’ appearance. (E) *35S::GmCLV3* plant with bolts initiated from primary as well as axillary meristems; no siliques were formed from the plant. (F) Wild-type flower. (G) Defective flower of *35S::GmCLV3* with missing carpel. (H) Phenotypes of *35S GmCLV3* in *clv3-2* background with normal shaped buds shown in the inset. (I) Three individual *clv3-2* plants with enlarged floral buds (inset). (J) Siliques of *35S::GmCLV3a* in *clv3-2* background (left) and club-shaped silique of *clv3-2* (right). Bar, 1 mm (A–C, F, G, J), 1 cm (D, E, H, I) (this figure is available in colour at *JXB* online).

The independent *Arabidopsis* transgenic lines successfully transformed with *35S::GmCLV3a* displayed significant developmental defects (Fig. 4). The SAM stopped initiating organ after the appearance of first leaves (Fig. 4B and C). While strong *35S::GmCLV3* lines died prematurely (35 out of 47 independent lines), moderate to weak transgenic lines (12 out of 47 independent lines) overcame the initial meristem arrest and subsequently produced misshapen leaves with irregular phyllotaxis (Fig. 4D). The mortality observed is in line with the reported ectopic expression analysis of *CLV3* and several members of the CLE family (Brand *et al.*, 2000; Strabala *et al.*, 2006). The ability of several *35S::GmCLV3a* lines to overcome the initial meristem arrest is consistent with reports that long-term expression of high levels of CLV3 can result in the reactivation of *WUS* expression (Muller *et al.*, 2006). Surviving plants displayed significant developmental timing delays as they remained vegetative for close to 2 months after germination (Fig. 4D) while first floral bud was visible with wild-type plants 5 weeks after germination under these growth conditions (data not shown). Bolting was eventually initiated almost simultaneously from primary as well as axillary meristem (Fig. 4E). As these plants developed further into the reproductive stage, they produced flowers with missing carpel and fewer anthers than normal. As a result, no siliques were formed from any of these lines (Fig. 4E). These phenotypes are reminiscent of the gain-of-function analysis with *Arabidopsis* CLV3 (Brand *et al.*, 2000).

### Ectopic expression of *GmCLV3* overrides *Arabidopsis clv3* mutant phenotypes

The *clv3-2* mutant is characterized by enlarged vegetative meristem and floral buds, and with short, club-shaped siliques (Clark *et al.*, 1995). To further confirm a conserved function of the *GmCLV3* with its *Arabidopsis* counterpart, the construct (35S::GmCLV3a) was transformed into *clv3-2* plants (Clark *et al.*, 1995). The overexpression of *GmCLV3a* in *clv3-2* mutant lines rescued the phenotype (Fig. 4H–J) and most notably resulted in the generation of normally shaped siliques (Fig. 4J).

## Discussion

This study has identified the *Arabidopsis CLV3* counterpart in a major legume crop, soybean. Intriguingly, unlike in *Arabidopsis* whereby *CLV3* is expressed in stem cells of all three outermost layers (L1–L3), *GmCLV3* is expressed deeper in the meristem beneath the fourth layer and coinciding with *GmWUS* expression in the organizing centre (Fig. 3). Subsequent investigation with an alternative stem cell marker (*GmLOG1*) reveals a different expression pattern of *GmLOG1* to that of *GmCLV3*. Based on the expression of *GmLOG1*, the location of soybean stem cells is likely to be within L2 to L4 layers of the central zone (Fig. 3D) and hence indicating *GmCLV3* is not a stem cell marker in soybean.

The markedly different expression of *GmCLV3* implicates its signalling mechanisms must have diverged from that of *Arabidopsis* especially since *GmCLV1* is not detectable in the central zone of the SAM via RT-PCR analysis. The absence of *GmCLV1* in the SAM is further corroborated with previous microarray dataset derived from studies examining the expression profile of dissected soybean SAM as the expression of *GmCLV1* was identified as absent in the SAM microarray dataset (Haerizadeh *et al.*, 2009). This study also failed to localize the expression of *GmCLV1* using *in situ* hybridization analysis. To date, two other pathways involving CLV2/CORYNE heterodimer or a receptor-like protein kinase TOAD2 homomer have been found to act as receptors for CLV3 ligand in parallel to the CLV1 pathway in *Arabidopsis* to negatively regulate the expression of *WUS* (Muller *et al.*, 2008; Kinoshita *et al.*, 2010). GmCLV3 could thus act through similar CLV2/CORYNE and/or TOAD2-associated pathways in soybean.

Studies in *Arabidopsis* have demonstrated the movement of the CLE peptide of CLV3 and WUS in the SAM, although the relevance of this movement is unclear (Nimchuk *et al.*, 2011; Yadav *et al.*, 2011). It is likely that the overlapping expression of *GmCLV3* and *GmWUS* in soybean reflects the situation in many plants. Nevertheless, future studies examining the receptor systems of GmCLV3 shall shade light on the biological relevance of this novel spatial expression pattern of *GmCLV3*.

**Fig. 5.**
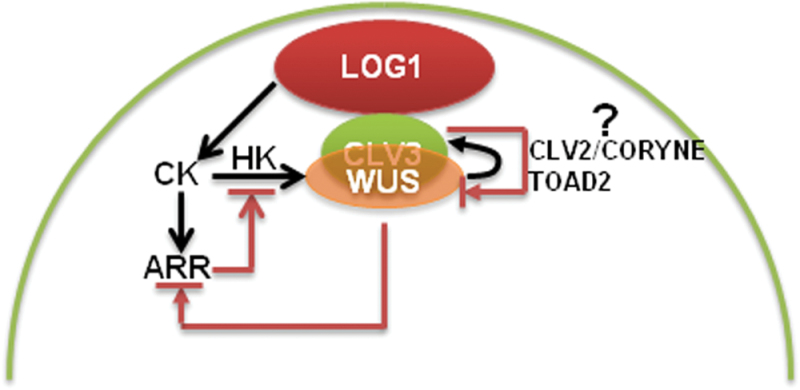
Regulatory networks in the soybean shoot apical meristem. A hypothetical model showing the action of cytokinin, WUS, and CLV3 in concert with other factors (described in the text) in regulating the stem cell population within the shoot apical meristem in soybean. Histidine kinase (HK) functions as a receptor for cytokinin (CK) while type-A *ARABIDOPSIS RESPONSE REGULATOR (ARR)* acts as a negative regulator for cytokinin signalling (Leibfried *et al.*, 2005; Gordon *et al.*, 2009) (this figure is available in colour at JXB online)

Stem cells in the SAM as well as axillary meristem in soybean are associated with the expression of *GmLOG1*. In fact, *GmLOG1* expression marks the initials for axillary meristem at the leaf axil and, as far as is known, this spatial expression pattern of *GmLOG1* has not been reported before (Kurakawa *et al.*, 2007; Aggarwal *et al.*, 2010). The current study thus implicates cytokinin in early axillary meristem development, possibly by inhibiting cell growth or conferring meristematic competency to axillary initials. Furthermore, given that neither *GmWUS* nor *GmCLV3* expression is detected in these axillary initials or the surrounding neighbouring cells but only in axillary meristems, it is conceivable that cytokinin may be the primary signal that initiates and sustains an environment promoting stem cell organization. In *Arabidopsis*, cytokinin has been implicated in specifying a spatial WUS-expressing domain which then promotes stem cell number in the overlaying region acting in concert with cytokinin receptors and response regulators (Gordon *et al.*, 2009). We have recently carried out transcriptome sequencing of total RNA derived from micro-dissected SAM (Wong *et al.*, 2013). An examination of the resulting dataset reveals the expression of several putative cytokinin receptors or regulators in the SAM (Supplemental Table 1). Similar histidine kinases and response regulators could function as receptors or regulators for cytokinin. They could thus act in a similar signalling feedback loops that operate and may take precedence over *GmCLVs* in the control of stem cell number within soybean SAM (Fig. 5).

Meanwhile, the absence of *GmLOG1* from the outermost layer of the soybean meristem is noteworthy. It is possible that true stem cell marker is yet to be uncovered and the expression of *GmLOG1* in stems cells located in the L2–L4 layer may serve as a sufficient source of cytokinin for it to exert its roles in the SAM.

Despite the novel expression pattern, *GmCLV3* seems to have the capacity to function as *CLV3* when ectopically expressed in *Arabidopsis* and *clv3-2* mutant. This implies that the GmCLV3 ligand could interact with the *Arabidopsis* CLV3 receptor(s) and, hence, consistent with previous studies reporting the capacity of CLE ligands for functional redundancy (Wang *et al.*, 2005; Strabala *et al.*, 2006).

In summary, this work successfully isolated the soybean *CLV3* orthologue (*GmCLV3*) and showed its markedly different spatial expression pattern from its *Arabidopsis* counterpart. Though ectopic expression studies of *GmCLV3* in *Arabidopsis* and mutant complementation work suggest largely similar functional characteristics, the novel expression pattern of *GmCLV3* and the lack of *GmCLV1* expression in the central zone implicate divergence in the CLV pathway in soybean. Moreover, this spatial localization study of *GmLOG1* implicates a primary role of cyokinin in initiating and sustaining stem cell population within the meristem.

## Supplementary material

Supplementary data are available at *JXB* online.

Supplementary Fig. 1. Alignment analysis of *GmCLV3a* and *GmCLV3b*

Supplementary Table 1. Expression levels of putative cytokinin receptors and response regulators in soybean shoot apical meristem

Supplementary Data
