# Supplementary material for: Spatial expression of CLAVATA3 in the shoot apical meristem suggests it is not a stem cell marker in soybean
Source: J Exp Bot. 2013 Oct 31;64(18):5641–9. doi: 10.1093/jxb/ert341 (PMC3871822; doi:10.1093/jxb/ert341)
Supplement: Supplementary Data [file supp_64_18_5641__index.html]

Spatial expression of CLAVATA3 in the shoot apical meristem suggests it is not a stem cell marker in soybean — Spatial expression of CLAVATA3 in the shoot apical meristem suggests it is not a stem cell marker in soybean — Supplementary Data 

# Spatial expression of *CLAVATA3* in the shoot apical meristem suggests it is not a stem cell marker in soybean

## Supplementary Data

Data files

**Files in this Data Supplement:**

- Supplementary Data - Supplementary Data
