# Supplementary material for: Spatial expression of CLAVATA3 in the shoot apical meristem suggests it is not a stem cell marker in soybean
Source: J Exp Bot. 2013 Oct 31;64(18):5641–9. doi: 10.1093/jxb/ert341 (PMC3871822; doi:10.1093/jxb/ert341)
Supplement: Supplementary Data [file supp_ert341_jexbot102327_file001.pdf]

Spatial expression of *CLAVATA3* in the shoot apical meristem suggests it is not a stem cell marker in soybean

Chui E Wong, Mohan B Singh and Prem L Bhalla*

Plant Molecular Biology and Biotechnology Group, ARC Centre of Excellence for Integrative Legume Research, Melbourne School of Land and Environment, The University of Melbourne, Parkville, Victoria 3010, Australia.

*To whom correspondance should be addressed. Email: premlb@unimelb.edu.au

Supplemental Figure 1. Alignment analysis of *GmCLV3a* and *GmCLV3b* with the start and stop codon highlighted. Underlined regions are the respective location of primer sites for GmCLV3a and GmCLV3b.

```
GmCLV3a         CTATAAATACGTGTATTACCCTATGATCTTCACCACACAACATTACTTGTTCACCACCTT
GmCLV3b         CTATAAATACGTGCATAACCCCATACTCTTCTTCACAACACAGCACTTGTTCACCACCTG
                ************* ** **** **  *****  ****  ***  *************** 

GmCLV3a         GCTCTCCTTGCAACTTCCACCCCTACCACCTACCTTGATTCTCTTGAGACTTTTTTTCC
GmCLV3b         GCTATCCTTGCAACTTCCACCCCT---ACCTAGTTTGATTCTCT-AAGACCTTTTT-CC
                *** ********************   *****  **********  **** ***** **

GmCLV3a         AATGGCGTCAAAGTTTATCTTTACCACTGTGGTTTTACTTATGCTTTTGTGCTTGCTTCT
GmCLV3b         AATGGCATCAAAGTTTATCTTTACCACGGTGGTTTTAGTTATGCTTTTGTGCTTGCTTCT
                ****** ******************** ********* **********************

GmCLV3a         GATGAGGGAGCCTTCTGGTTGCGGTTCTGCGTATGAATGCTTTGGTGCTAATGCAGCTGA
GmCLV3b         TATGAGGGACCCTTCTGGTTGTGGTTCTGCATATGAATGCTTTGGTGCTAATGCAGCTGG
                 ******** *********** ******** **************************** 

GmCLV3a         TCTAAGGGACATTCCAAACAGGAAGGTGCTGTCTGTTTTGAAGGATAAGAAAACTAGTGC
GmCLV3b         TCTTACGGACATTCCAAACAGGAAGGTGCTGCCTGTTTTGAAGGATAAGAAAACTAGTGC
                *** * ************************* ****************************

GmCLV3a         TCTGAAGGCTAGTCTGCAAGGATCATCAAGCAACAAGTATGGTGAAAAGCCACTGAATTG
GmCLV3b         TCTGAAGGCTAGTCTGCGAGGATCATCAAGCAACAAGTATGGTGAAAAGCCACTGAATTG
                ***************** ******************************************

GmCLV3a         GGAGTTAAGGAAGGTTCCTTCTGGTCCAGATCCGCTGCATCATAATGGTGTCAACCCCAA
GmCLV3b         GGAGTTAAGGAAGGTTCCTTCAGGTCCAGATCCGCTGCATCATAACGGTGTGAACCCCAA
                ********************* *********************** ***** ********

GmCLV3a         AAAGCCTCAAACCCCTTAAACTCACCGATGGAACCTTCTTTGTGCTCTTGGCCTAGTATT
GmCLV3b         AAATCCTCAAACCCCTTAAGCTCACCTATGGAACCTGCTTTTTGCTCTTGGCCTAGTGTT
                *** *************** ****** ********* **** *************** **

GmCLV3a         AGTTATCAGTGTG--AACATCTACCAGCTTATGGCATAAAT------------
GmCLV3b         AGTTATCAGTGTGTGAACATCTACAAGCTTTTGGCATAAGTTTATTGAAAAAT
                *************  ********* ***** ******** *
```

Supplemental Table 1. Expression levels of GmCLV1 homeolog, GmWUS, and putative cytokinin receptors and response regulators in soybean shoot apical meristem (SAM). SAMs were dissected as detailed in Wong et al., 2009 (Wong *et al.*, 2009) and total RNA was extracted using Tri reagent (Sigma) according to manufacturer's instructions and with DNAse digestion step incorporated. The cDNA library preparation and sequencing was carried out by Beijing Genomics Institute (China) according to the manufacturer's instructions (Illumina HiSeq™ 2000 platform). Sequence reads were processed using SOAP2 (Li *et al.*, 2009) and the abundance for each gene was calculated and expressed in RPKM (Mortazavi *et al.*, 2008).

Gene ID	SAM	Annotation
**Glyma11g12190**	0.50	CLAVATA1
**Glyma12g04390**	0.04	CLAVATA1
**Glyma01g37190**	9.67	WUSCHEL
**Glyma01g04420.1**	1.05	ARR5 (ARABIDOPSIS RESPONSE REGULATOR 5)
**Glyma02g09450.1**	11.97	ARR2 (ARABIDOPSIS RESPONSE REGULATOR 2)
**Glyma02g47610.1**	17.99	AHK2 (ARABIDOPSIS HISTIDINE KINASE 2)
**Glyma04g06650.1**	14.08	ARR12 (ARABIDOPSIS RESPONSE REGULATOR 12)
**Glyma04g40100.1**	11.54	ARR17 (ARABIDOPSIS RESPONSE REGULATOR 17)
**Glyma05g28070.1**	10.26	AHK3 (ARABIDOPSIS HISTIDINE KINASE 3)
**Glyma06g06730.1**	30.45	ARR12 (ARABIDOPSIS RESPONSE REGULATOR 12)
**Glyma06g14750.1**	51.87	ARR17 (ARABIDOPSIS RESPONSE REGULATOR 17)
**Glyma07g08590.1**	3.07	ARR2 (ARABIDOPSIS RESPONSE REGULATOR 2)
**Glyma07g26890.1**	15.73	ARR2 (ARABIDOPSIS RESPONSE REGULATOR 2)
**Glyma07g37220.1**	25.69	ARR2 (ARABIDOPSIS RESPONSE REGULATOR 2)
**Glyma08g11060.1**	1.11	AHK3 (ARABIDOPSIS HISTIDINE KINASE 3)
**Glyma09g04470.1**	9.85	ARR2 (ARABIDOPSIS RESPONSE REGULATOR 2)
**Glyma09g14650.1**	17.71	ARR12 (ARABIDOPSIS RESPONSE REGULATOR 12)
**Glyma13g22320.1**	1.54	ARR12 (ARABIDOPSIS RESPONSE REGULATOR 12)
**Glyma14g01040.1**	11.66	AHK2 (ARABIDOPSIS HISTIDINE KINASE 2)
**Glyma14g12330.1**	2.21	AHK5 (ARABIDOPSIS HISTIDINE KINASE 5)
**Glyma15g15520.1**	9.47	ARR2 (ARABIDOPSIS RESPONSE REGULATOR 2)
**Glyma15g24770.1**	9.67	ARR12 (ARABIDOPSIS RESPONSE REGULATOR 12)
**Glyma17g03380.1**	24.33	ARR2 (ARABIDOPSIS RESPONSE REGULATOR 2)
**Glyma17g08380.1**	6.42	ARR18 (ARABIDOPSIS RESPONSE REGULATOR 18)
**Glyma17g33230.1**	1.08	ARR12 (ARABIDOPSIS RESPONSE REGULATOR 12)
